# Rotenone Susceptibility Phenotype in Olfactory Derived Patient Cells as a Model of Idiopathic Parkinson’s Disease

**DOI:** 10.1371/journal.pone.0154544

**Published:** 2016-04-28

**Authors:** M. Murtaza, J. Shan, N. Matigian, M. Todorovic, A. L. Cook, S. Ravishankar, L. F. Dong, J. Neuzil, P. Silburn, A. Mackay-Sim, G. D. Mellick, S. A. Wood

**Affiliations:** 1 Eskitis Institute for Drug Discovery, Griffith University, Brisbane, Queensland, Australia; 2 Wicking Dementia Research and Education Centre, University of Tasmania, Hobart, Tasmania, Australia; 3 Apoptosis Research Group, School of Medical Science, Griffith University, Southport, Queensland, Australia; 4 Asia-Pacific Centre for Neuromodulation, University of Queensland, Brisbane, Australia; Hokkaido University, JAPAN

## Abstract

Parkinson’s disease is a complex age-related neurodegenerative disorder. Approximately 90% of Parkinson’s disease cases are idiopathic, of unknown origin. The aetiology of Parkinson’s disease is not fully understood but increasing evidence implies a failure in fundamental cellular processes including mitochondrial dysfunction and increased oxidative stress. To dissect the cellular events underlying idiopathic Parkinson’s disease, we use primary cell lines established from the olfactory mucosa of Parkinson’s disease patients. Previous metabolic and transcriptomic analyses identified deficiencies in stress response pathways in patient-derived cell lines. The aim of this study was to investigate whether these deficiencies manifested as increased susceptibility, as measured by cell viability, to a range of extrinsic stressors. We identified that patient-derived cells are more sensitive to mitochondrial complex I inhibition and hydrogen peroxide induced oxidative stress, than controls. Exposure to low levels (50 nM) of rotenone led to increased apoptosis in patient-derived cells. We identified an endogenous deficit in mitochondrial complex I in patient-derived cells, but this did not directly correlate with rotenone-sensitivity. We further characterized the sensitivity to rotenone and identified that it was partly associated with heat shock protein 27 levels. Finally, transcriptomic analysis following rotenone exposure revealed that patient-derived cells express a diminished response to rotenone-induced stress compared with cells from healthy controls. Our cellular model of idiopathic Parkinson’s disease displays a clear susceptibility phenotype to mitochondrial stress. The determination of molecular mechanisms underpinning this susceptibility may lead to the identification of biomarkers for either disease onset or progression.

## Introduction

Parkinson’s disease is a complex age-related disorder, affecting approximately 2% of the population over 60 years [1]. The classical motor symptoms of Parkinson’s disease are rigidity, postural reflex impairment, resting tremor and bradykinesia. The main pathological hallmarks of Parkinson’s disease are the progressive loss of dopaminergic neurons from the pars compacta of the substantia nigra and the presence of cytoplasmic inclusions called Lewy bodies. Parkinson’s disease is now recognized as a systemic disease impacting tissues within and outside the central nervous system [2–5].

Approximately 90% of Parkinson’s disease cases are idiopathic, of unknown origin, while 10% have a familial origin [6,7]. The separation between idiopathic and familial cases of Parkinson’s disease is becoming less distinct, with the identification of common pathways shared between idiopathic and familial cases of Parkinson’s disease [8–15]. Extensive studies from genetic cellular and animal models of Parkinson’s disease implicate mitochondrial dysfunction, increased oxidative stress, impaired proteasomal degradation and calcium buffering as prominent contributors to the disease process and these bioenergetic deficits are not restricted to dopaminergic neurons [16–19].

In recent years, patient-derived cells have been used to generate disease-specific cellular models with varying degrees of success. In fibroblasts, derived from skin of idiopathic Parkinson’s disease patients diminished pyruvate utilization, reduced mitochondrial complex I activity and increased lipid peroxidation were observed, similar to post-mortem brain tissue [20–24]. Induced pluripotent stem cell (iPS) technology and the ability to differentiate reprogrammed cells into dopaminergic neurons represents a significant advancement in the field and it is a rapidly developing *in vitro* model to study disease mechanisms [25]. Although induced pluripotent stem cells have been derived from idiopathic Parkinson’s disease patients, the first study using dopaminergic neurons derived from iPS cells reported the lack of conspicuous disease-related phenotypes [26]. In contrast, a later study reported that prolonged culture of iPS-derived dopaminergic neurons *in vitro* results in spontaneous disease pathology, particularly, increased susceptibility to neurodegeneration and defective autophagy [27]. However, the variability in the reprogramming process, epigenetic status between cell lines and heterogeneity of neural differentiation [26,28] still raises some concerns about the use of reprogramming in the modelling of human diseases with complex aetiology.

Physiologically relevant and easily accessible cellular models of idiopathic Parkinson’s disease are essential for understanding disease pathology and for high throughput screening of drug candidates. The underlying molecular and cellular mechanisms of idiopathic forms of Parkinson’s disease are not well defined. We have previously reported that olfactory neurosphere-derived cells (ONS) obtained from the olfactory mucosal epithelium of idiopathic Parkinson’s disease patients display metabolic and molecular differences compared to age and gender-matched healthy controls [29,30]. Interestingly, we also identified a dysregulation in the stress-response pathway NRF2 in patient-derived cells. There is ample literature highlighting the role of cellular stress in the progression of Parkinson’s disease. The primary implication from these studies is that patient cells, in particular but not exclusively, dopaminergic neurons are less capable of mounting a robust stress response [10,31–34]. We hypothesized that patient-derived cells deal with cellular stress in an atypical fashion. The main aim of our study was to investigate whether bioenergetic deficits associated with Parkinson’s disease and reported at a central level can be detected in ONS cells derived from idiopathic Parkinson’s disease patients. To investigate this, we assayed extrinsic stressors affecting mitochondrial complex, lysosomes, proteasome, endoplasmic reticulum, oxidative stress and DNA damage. Our results reveal an endogenous deficit in mitochondrial complex I in patient-derived cells and an increased susceptibility of patient-derived cells to rotenone-induced mitochondrial complex I inhibition and H_2_O_2_ induced oxidative stress. We further characterized the cell pathology underlying the sensitivity of patient-derived cells to rotenone and identified that this was partly associated with heat shock protein 27 (HSP27) levels in the cell. Finally, we determined that exposure to mitochondrial complex I inhibitor, rotenone, affects the transcriptional responses of patient-derived cells differently compared to control-derived cells.

In summary, based on comparison of multiple patient-derived and control-derived cell lines, we identified disease-specific differences in response to cellular stress that result in increased cell apoptosis, we also identified an endogenous deficit in mitochondrial complex I in idiopathic Parkinson’s disease and this may represent a point of convergence of genetic and idiopathic forms of the disease.

## Materials and Methods

### Ethics statement

All donor tissue and information was obtained with informed and written consent of the participants. All procedures were in accordance with National Health and Medical Research Council Code of Practice for Human Experimentation and approved by the Griffith University Human Experimentation Ethics Committee.

### Participants and olfactory biopsies

Patients with idiopathic Parkinson’s disease (N = 19), genetic Parkinson’s disease (N = 4) MND (N = 5), HSP (N = 5) were recruited from consumer groups and through research participant registers maintained by the Queensland Parkinson’s Project and the Queensland Centre for Mental Health research. Controls (N = 20) were recruited from the general population. The patients were diagnosed by a movement disorders neurologist according to UK Brain Bank criteria. The same questionnaire was completed by patients and healthy controls. Olfactory biopsies were obtained by a specialist otorhinolaryngologist according to previously published protocols [35,36]. Details of age, gender and cell line ID are given in supplementary S1 Table

### Cell Culture

Olfactory neurosphere-derived cells (ONS) derived from patients with Parkinson’s disease are referred to as “Patient-derived” cells. Cells derived from healthy control subjects are referred to as “Control-derived” cells. Cell lines from patient and healthy control donors were established as previously described [30]. Frozen aliquots of patient-derived and control-derived cells were thawed and cultured in DMEM/F12 (Invitrogen) supplemented with 10% fetal bovine serum FBS (Invitrogen). The medium was refreshed every other day until the cells reached confluency. All assays were performed with cells cultured for similar periods after nasal biopsy (under 10 passages from the initial plating).

### Determination of specific stressor concentration and treatment of ONS cells

Control-derived and patient-derived cells were exposed to the following inhibitors: epoxomicin, chloroquine, camptothecin, rotenone, tunicamycin and hydrogen peroxide. A titration was performed to determine sub-lethal concentrations of each inhibitor, which led to the loss of approximately 20% of the cells over 48 hours (determined by CyQUANT assay). Cells were enzymatically harvested using TrypLE Express (Invitrogen) and resuspended in DMEM/F12 supplemented with 10% FBS (GibcoBRL) and 2,500 cells were seeded into each well of a 96-well plate (Nunc). After incubating at 37°C, 5% CO_2_ for 12 hours, the medium was replaced with medium containing 50 nM rotenone, 80 μM H_2_O_2_, 10 nM epoxomicin, 40 μM chloroquine, 10 nM camptothecin and 40 nM tunicamycin. The cells were exposed to the stressors for up to 120 hours with DNA content measured every 24 hours.

### CyQUANT (DNA content) assay

CyQUANT assay is based on the measurement of cellular DNA content via fluorescent dye binding to cellular DNA. CyQUANT assay was carried out as per manufacturer’s instructions (Invitrogen). Briefly, ONS cells in black-walled 96 or 384 well culture plates (Nunc) were washed twice with HBSS buffer (Invitrogen) and either 50 μL or 12.5 μL of reaction mixture containing (1X CyQUANT dye reagent and 1X dye delivery reagent) was added in each well and incubated at 37°C for 90 min, then the fluorescence intensity of each sample was measured using a Synergy II plate reader (BioTek) with excitation at ~485 nm & emission detection at ~530 nm.

### Apoptosis (Caspase 3/7 activity) assay

The apoptosis assay based on the measurement of Caspase 3/7 activity was performed according to the manufacturer’s instructions (Promega). Briefly, 100 μL of Apo-ONE Caspase 3/7 reagent (Promega) was added into each well of white-walled 96-well (Nunc) plate and incubated on a shaker at 300–500 rpm for 5 min followed by one hour incubation at room temperature. The luminescence of each sample was measured using a luminometer.

### Immunoblot

Cells, seeded and incubated in 75 cm^2^ flasks (Nunc), were scraped in 200 μl of lysis buffer (Tris 40 mM pH 7.5, KCl 150 mM, EDTA 1 mM, Triton X-100 1%) containing a protease inhibitor mixture (Roche Molecular Biochemicals). Protein concentration was determined using BCA protein assay kit (Pierce). 2.5 μg of each sample was electrophoresed in a 4–12% polyacrylamide minigel. The proteins were transferred onto nitrocellulose membranes, according to the manufacturer’s instructions (Invitrogen). The membranes were blocked in 5% non-fat dry milk in phosphate buffer saline for 1 hour at room temperature and then incubated with (1:1000) anti-HSP27 or anti-α-actin at 4°C overnight. The membranes were washed three times with 0.1% Tween 20 in PBS and then incubated with anti-rabbit IgG or anti-mouse IgG conjugated to HRP (1:5000) (Millipore). The immunocomplexes were visualised by the ECL chemiluminescence method (Millipore) and a digital imaging station (VersaDoc, BioRad).

### Nucleofection

Control-derived and Parkinson’s disease patient-derived cells were cultured in antibiotic-free growth medium in 75 cm^2^ flasks (Nunc). 375,000 cells in suspension were transfected by nucleofection with pEGFP-HSP27 or pMax-GFP using the Amaxa Nucleofector Kit (Lonza) as per manufacturer’s instructions. As a sham control, 375,000 cells were subjected to same nucleofection conditions without any plasmid. pEGFP-HSP27 wt FL was a gift from Andrea Doseff (Addgene Plasmid #17444). Transfection efficiency was monitored by GFP fluorescence. After 72 hours, cells were treated with Rotenone (50 nM) or DMSO (vehicle– 0.05%) for 48 hours. Cells were harvested and counted using Countess II (Life Technologies) as per manufacturer’s instructions.

### Isolation of Mitochondria

Mitochondria were isolated from ONS cells using the magnetic bead mitochondrial isolation kit (Miltenyi Biotec) as per manufacturer’s instructions and using the standard method. For standard isolation, half million ONS cells were harvested and gently homogenized in 1 mL of chilled mitochondrial isolation buffer (250 mM sucrose, 2 mmole/l Hepes, 0.1 mmole/l EGTA, pH = 7.4) with a needle homogenizer (3 mL syringe with 0.6 × 32 mm needle 20 times). The homogenate was then centrifuged at 600 × g for 10 min at 4°C to remove the cell debris. The supernatant was further centrifuged at 14,400 × g for 10 min and the pellet containing mitochondria was resuspended in 150 μL hypotonic buffer (25 mM potassium phosphate with 5 mM MgCl2, pH = 7.2) and the concentration of mitochondria was determined by BCA protein assay kit (Pierce).

### Mitochondrial complex I, complex II and citrate synthase activity assay

Complex I activity was measured in 95 μL of 50 mmole/l KPi (pH = 7.4) containing 0.75 mmole/l NADH, 20 μg/mL Coenzyme Q1 and 2 mmole/l KCN. The reaction was started by the addition of 5 μL of mitochondria in a 96-well plate. The assay rate was monitored spectrophotometrically by the decrease in absorbance at 340 nm for 20 min. The rotenone insensitive complex I activity was determined simultaneously by measuring a sample with 25 μmole/l rotenone added to the reaction mixture. Rotenone-specific complex I activity was calculated as the total activity minus the rotenone-insensitive activity. The activity unit is expressed as n mole NADH/min/mg.

Complex II activity was measured in 480 μL of 50 mM KPi (pH = 7.4) containing 10 mmole/l sodium succinate, 25 μmole/l rotenone and 2 mmole/l KCN. 20 μL of mitochondria mixed with 480 μL reaction mixture in 0.7 mL quartz cuvette was incubated at room temperature for 10 min to activate complex II. The reaction was started by the addition of 10 μL of 2.5 mM Coenzyme Q1 and the assay rate was monitored spectrophotometrically by the decrease in absorbance at 280 nm for 10 min. The activity unit is expressed as n mole CoQ1/min/mg.

Citrate synthase activity was measured in 180 μL of 50 mM PBS (pH = 7.4) containing 0.3 mmole/l Acetyl-coezyme A, 0.5 mmole/l Oxaloacetate and 0.1 mmole/l 5,5'-dithiobis-(2-nitrobenzoic acid) (DTNB). The reaction was started by the addition of 20 μL of mitochondria in a 96-well plate. The assay rate was monitored spectrophotometrically by the increase in absorbance at 412 nm for 10 min. The activity unit is expressed as nmole/min/mg.

### Data Analysis

Statistical analysis was performed using GraphPad Prism 5 and differences of patient-derived cells versus control-derived cells, or between groups in stressor experiments were assessed by two-way ANOVA. Data are presented as mean ± SEM or mean ± SD. α<0.05 was considered significant.

### Microarray hybridisation and analysis

8 control and 8 patient-derived ONS cell lines were treated with either DMSO or 50nM Rotenone for 48 hours and profiled using the Illumina Beadarray Ref8v2 Chips. Microarray data are available in the ArrayExpress database (http://www.ebi.ac.uk/arrayexpress) under accession number E-MTAB-4164.

RNA was extracted using commercially available kits (Qiagen) and total RNA was reverse transcribed using SuperScript III first strand synthesis system (Invitrogen). Data processing and analysis was performed as reported previously [29].

## Results

### Patient-derived cells are differentially sensitive to mitochondrial and oxidative stress

To determine if ONS cells derived from idiopathic Parkinson’s disease patients displayed differential cytological sensitivity, we exposed patient-derived and control-derived cells to various cell stressors. These included inhibitors of the mitochondrial complex I (rotenone), oxidative stress (hydrogen peroxide), proteasome (epoxomicin), lysosome (chloroquine), DNA topoisomerase I (camptothecin) and inducers of the unfolded protein response (tunicamycin). After treatment, the number of viable cells was assayed using CyQUANT, which measures DNA content and is directly proportional to cell number (S1 Fig). A titration was performed to determine the compound concentration, which led to the loss of approximately 20% of the cells over 48 hours (S2 Fig). It was reasoned that this level of loss might accentuate the reproducible differences observed between patient-derived and control-derived cells. Using too high or too low a concentration would mask any disease-specific differences. Cells were then exposed to the compound at the beginning of each culture and cell number measured every 24 hours for 5 days.

**Fig 1:**
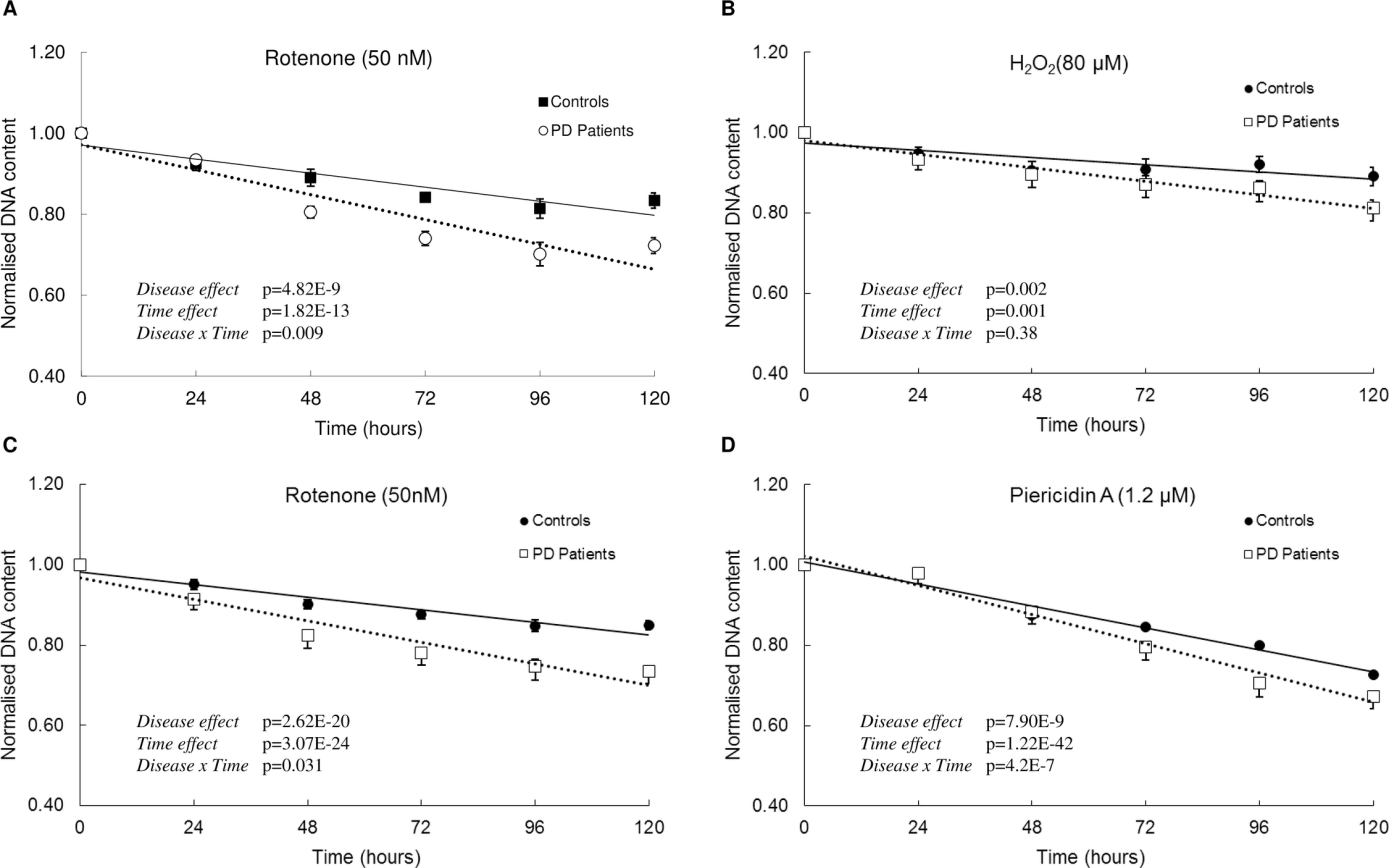
Effect of rotenone, hydrogen peroxide and piericidin A in control and patient-derived cells. Cells were exposed to (A) 50 nM Rotenone (controls n = 8, patients n = 8) (B) 80 μM Hydrogen Peroxide (C) 50 nM Rotenone (D) 1.2 μM Piericidin A. DNA content was determined every 24 hours by CyQUANT assay. Controls n = 8, patients n = 8 for A, B and D. Controls n = 19, patients n = 19 for C. Data are represented as Mean ± SEM, solid line represents linear fit for controls and dotted line represents linear fit for patients. Graphs are annotated with p-values from a two-way ANOVA.

We observed fewer patient-derived cells following exposure to rotenone (50 nM) and hydrogen peroxide (80 μM) compared to control-derived cells (Fig 1A and 1B). The differential effect of rotenone on patient-derived cells was evident from 48 hours until completion of the experiment at 120 hours (percentage change in patient-derived cells at each time point was 24 hours = 0.97%, 48 hours = -9.5%, 72 hours = -12.02%, 96 hours = -13.84% and 120 hours = -13.43%). There was a significant interaction between time of rotenone treatment and disease-status (time x disease, p<0.05). The differential effect of hydrogen peroxide was evident at 96 and 120 hours (percentage change in patient-derived cells at 24 hours = -1.44%, 48 hours = -1.28%, 72 hours = -4.14%, 96 hours = -6.26% and 120 hours = -8.84%). Given the volatile nature of hydrogen peroxide, and that it was only added once, at the beginning of the experiment, we measured its concentration in our system over time. Interestingly, although a differential effect was only apparent after 96 hours, the hydrogen peroxide was completely depleted within an hour of its addition at the beginning of the experiment (S3 Fig).

**Fig 2:**
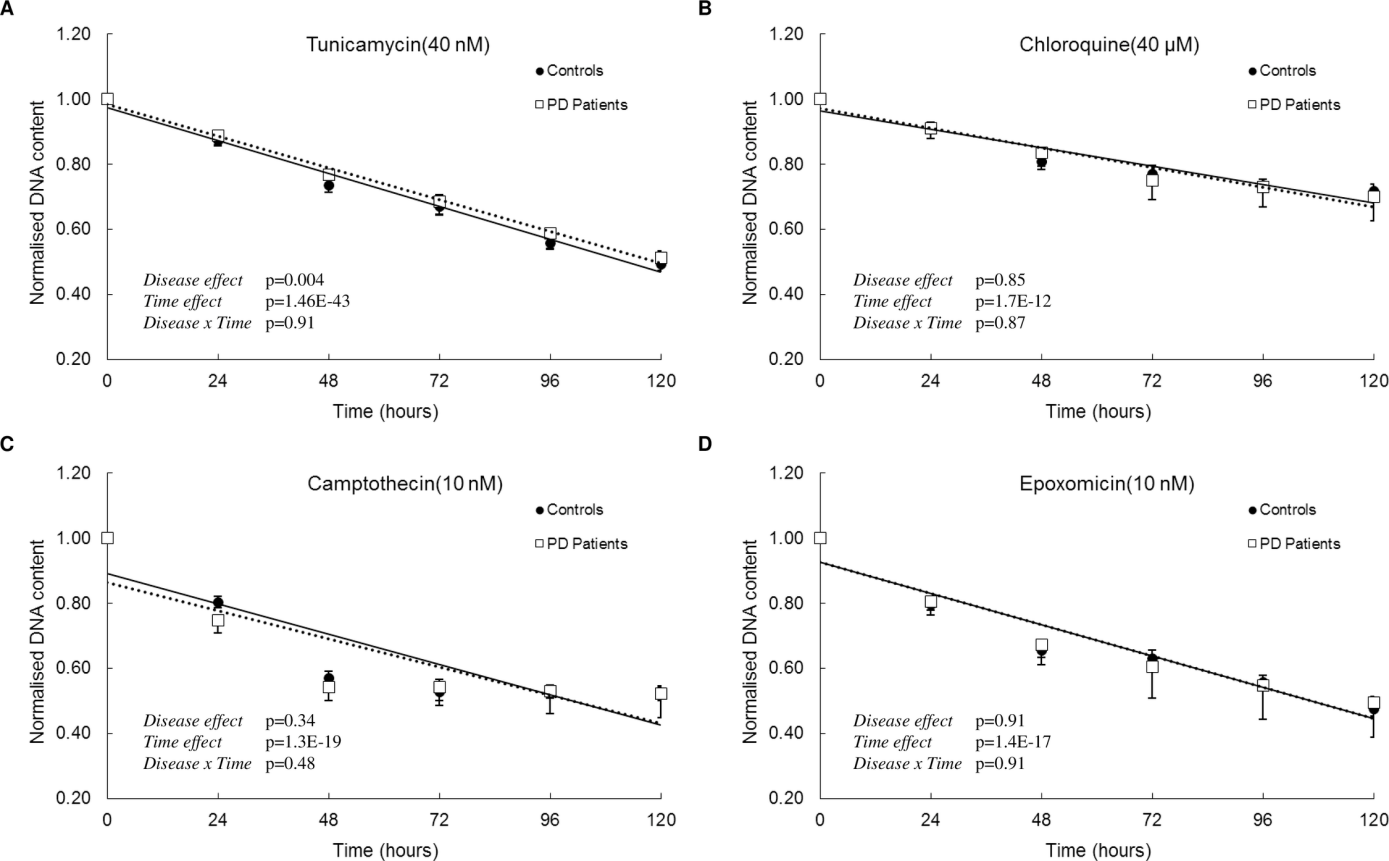
Effect of tunicamycin, chloroquine, camptothecin and epoxomicin in control and patient-derived cells. Cells were exposed to (A) 40 nM Tunicamycin (B) 40 μM Chloroquine (C) 10 nM Camptothecin and (D) 10 nM Epoxomicin over 120 hours in culture. DNA content was determined every 24 hours by CyQUANT assay. Controls n = 8, patients n = 8. Data are represented as Mean ± SEM, solid line represents linear fit for controls and dotted line represents linear fit for patients. Graphs are annotated with p-values from a two-way ANOVA.

To determine if sensitivity to rotenone was a common characteristic of patient-derived cells, we repeated the experiment on a larger sample of patient-derived (N = 19) and control-derived cells (N = 20). There was a significant interaction between time of rotenone treatment and disease-status (time x disease, p<0.05). The percentage change in patient-derived cells at each time point was -3.86% at 24 hours, -8.64% at 48 hours, -10.77% at 72 hours, -11.86% at 96 hours and -13.46% at 120 hours. This increased sample size confirmed the differential decrease in cell number in patient-derived cells following exposure to rotenone (Fig 1C). When ONS cells were exposed to an independent chemical complex I inhibitor, Piericidin A (1.2 μM) we observed patient-derived cells were more sensitive (24 hours = 0.14%, 48 hours = 1.09%, 72 hours = -5.98%, 96 hours = -11.78% and -7.45% at 120 hours) than control-derived cells (Fig 2D). There was a significant interaction between time of piericidin A treatment and disease-status (time x disease, p<0.0001). We also observed that patient-derived cells were slightly less vulnerable to tunicamycin treatment (24 hours = 1.8%, 48 hours = 4.4%, 72 hours = 2.09%, 96 hours = 5.44% and 120 hours = 3.82%) than control-derived cells, at least in this cohort (n = 8 versus 8) (Fig 2A). Patient-derived cells and control-derived cells responded similarly after exposure to epoxomicin, chloroquine, camptothecin. (Fig 2B–2D, percentage change at 120 hours for all 3 stressors was less than 4%). The percentage change in patient-derived cells after exposure to epoxomicin, chloroquine, camptothecin at 24 hours, 48 hours, 72 hours, 96 hours and 120 hours is presented in supplementary S2 Table. These data demonstrate that ONS cells derived from idiopathic Parkinson’s disease patients reveal disease-specific differences in response to cell stressors, especially oxidative stress.

### Rotenone induces apoptosis in Patient-derived cells

**Fig 3:**
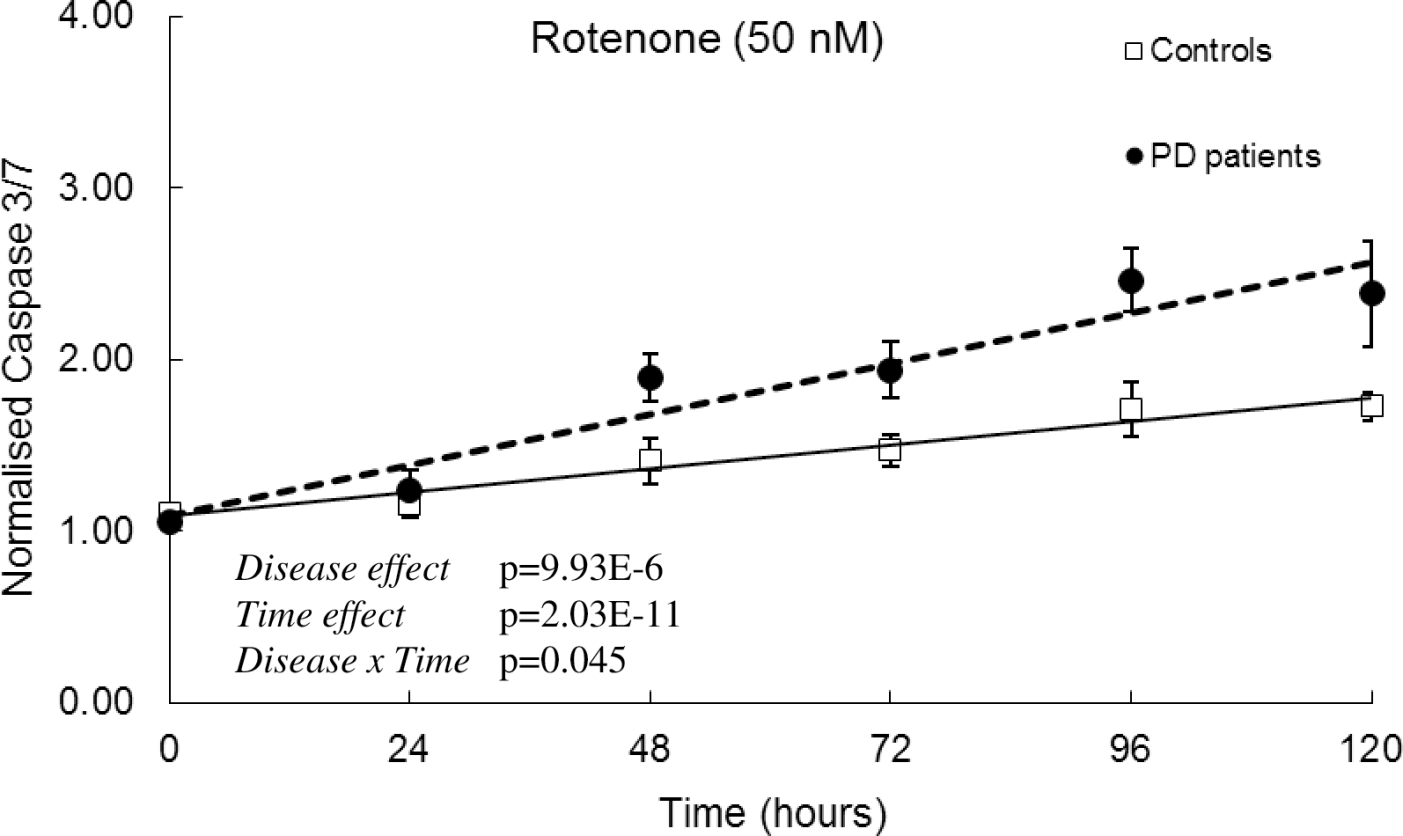
Increased apoptosis in patient-derived cells after exposure to rotenone. (A) Cells were exposed to 50 nM Rotenone and Caspase3/7 levels were determined every 24 hours for 120 hours in culture (n = 8 control vs. n = 8 patient-derived). Data are represented as Mean ± SEM, solid line represents linear fit for controls and dotted line represents linear fit for patients. Graphs are annotated with p-values from a two-way ANOVA.

To test whether patient-derived cells were more likely to undergo apoptosis in response to rotenone, we measured activated caspase 3/7 levels. We observed a time-dependent increase (24 hours = 6.5%, 48 hours = 34.33%, 72 hours = 31.93%, 96 hours = 43.5% and 120 hours = 37.68%) in caspase 3/7 levels in both control-derived and patient-derived cells. However, the patient-derived cells showed higher caspase 3/7 levels compared to control-derived cells and this difference was significant and indicative of increased apoptosis (Fig 3). The increased apoptosis and decreased viability of rotenone-exposed patient cells was confirmed in several independent apoptosis assays (S4 Fig).

**Fig 4:**
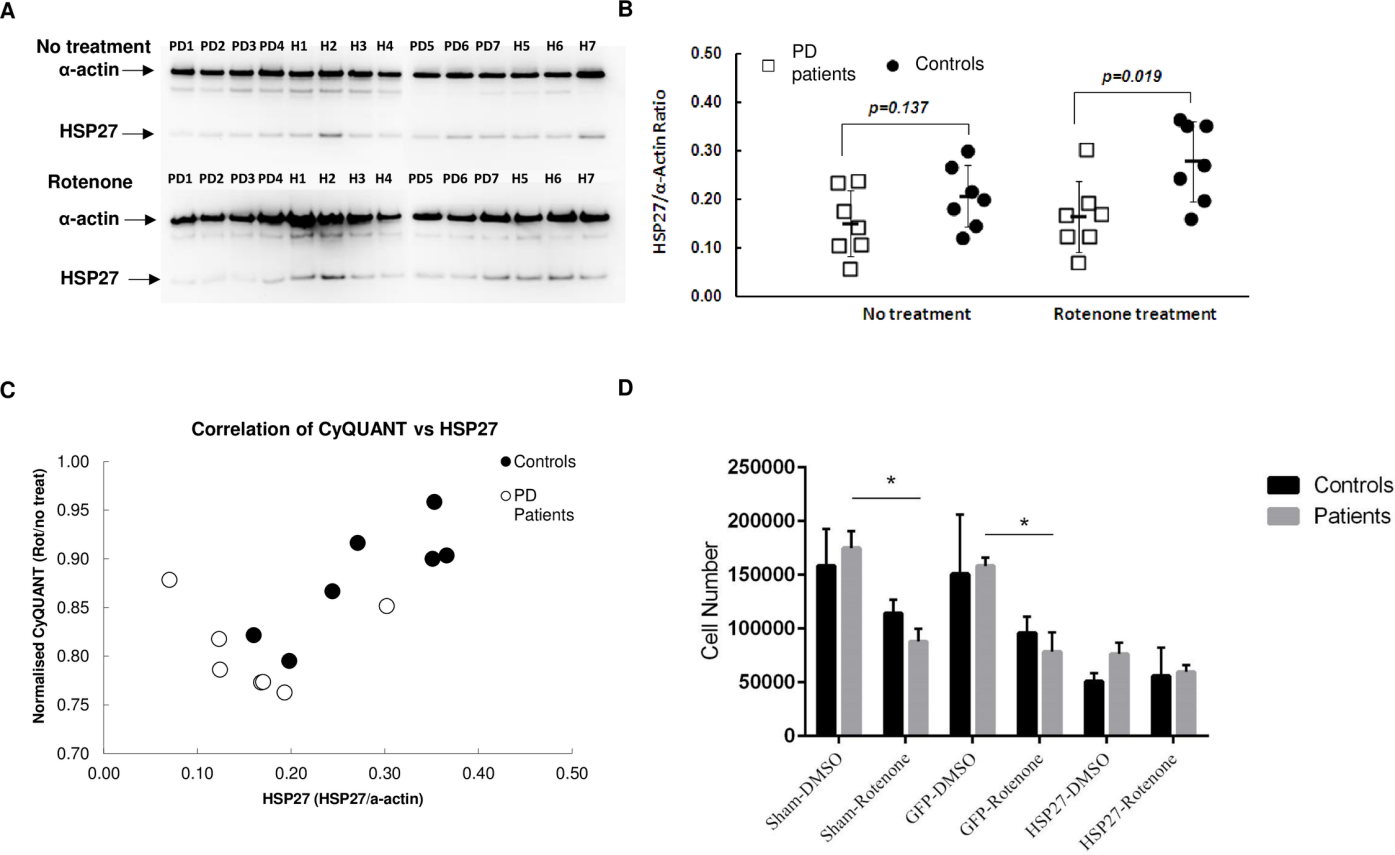
HSP27 protein levels in control and patient-derived cells. (A) Cells were exposed to 50 nM Rotenone for 48 hours. Total protein lysates from 7 control and 7 patient-derived cells were analysed by western blotting to detect endogenous HSP27 protein levels. α –Actin was used as a loading control. (B) Quantitative representation of HSP27 protein levels in untreated and Rotenone treated ONS cells relative to α –Actin levels. (C) Correlation analysis between normalized CyQUANT values (rotenone treated/no treatment) and HSP27 protein levels. The Pearson’s correlation coefficient for control cells is r = 0.84, p<0.05 and patient-derived cells is r = -0.09, p = 0.84) (D) Control and patient-derived cells were nucleofected with no plasmid, GFP-expressing plasmid and HSP27-GFP plasmid and cell number was determined after DMSO and 50 nM rotenone treatment. Controls n = 3 and patients n = 3, data are presented as Mean ± SEM * p<0.05

To investigate the underlying molecular basis of increased apoptosis, we screened a human apotosis protein array of 35 proteins (S8 Fig). Given the cytoprotective effect of HSP27 in response to rotenone-induced stress, we measured HSP27 protein levels in control-derived and patient-derived cells before and after rotenone treatment. This analysis indicated that while HSP27 protein levels increased in control-derived cells following exposure to rotenone, there was no increase in HSP27 levels in patient-derived cells and a significant deficit following rotenone exposure (Fig 4A and 4B). Interestingly, we observed a significant positive correlation (r = 0.84, p<0.05) between HSP27 protein levels and cell number in control-derived but no correlation (r = -0.09, p = 0.84) in patient-derived cells (Fig 4C).

To further characterize the role of HSP27 in rotenone-induced sensitivity, we nucleofected control-derived and patient-derived cells with HSP27-expressing plasmid and treated the cells with rotenone. GFP-expressing plasmid and sham nucleofections were used as experimental controls.

In control-derived cells, transfected with (i) no plasmid (sham), (ii) GFP-expressing plasmid or (iii) HSP27-expressing plasmid, and treated with rotenone, we did not detect a significant difference in cell number compared to transfected control-derived cells treated with vehicle only (DMSO).

Consistent with our results from the CyQUANT assay, in patient-derived cells, transfected with (i) no plasmid (sham), (ii) GFP-expressing plasmid and treated with rotenone, we observed a significant reduction in cell number compared to transfected patient-derived cells treated with vehicle only (DMSO). Unexpectedly, in both control-derived and patient-derived cells, we observed fewer cells after transfection with HSP27-expressing plasmid compared to the control transfections. Despite this, we observed that overexpression of HSP27 attenuates the susceptibility of patient-derived cells to rotenone treatment [percentage change in patient-derived cells compared with control-derived cells after (i) sham nucleofection + Rotenone -22.8%, (ii) GFP nucleofection + Rotenone -18.23%, (iii) HSP27 nucleofection + Rotenone 7.06%] (Fig 4D).

### Parkinson’s disease patient-derived cells show decreased mitochondrial complex I activity

**Fig 5:**
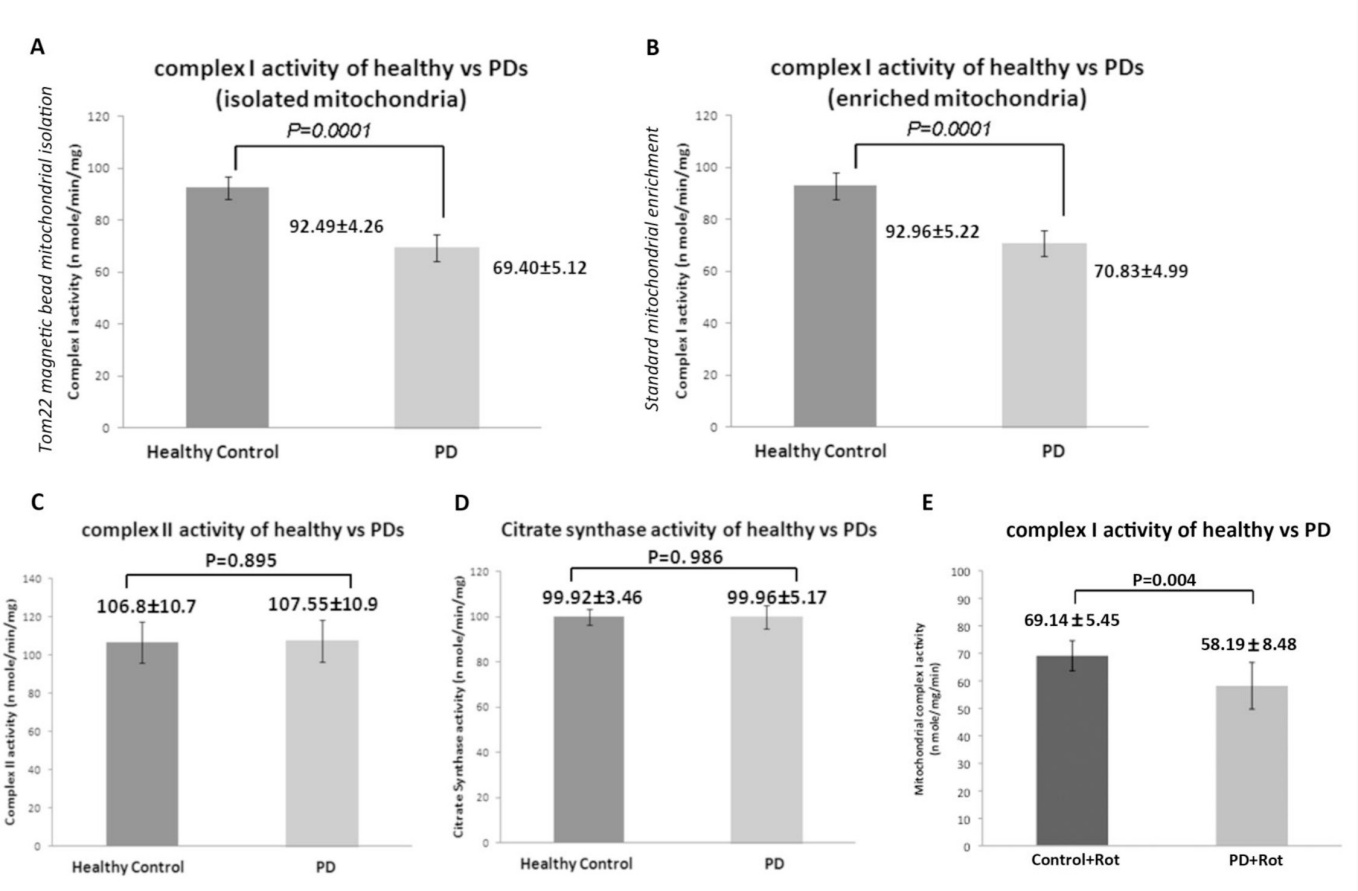
**Differences in the mitochondrial respiratory complexes between control and patient-derived cells** (A) Complex I activity levels in mitochondria isolated from cells using Tom22 magnetic beads. (B) Complex I activity in mitochondria isolated from control and patient-derived ONS cells using standard enrichment protocol. (C) Mitochondrial complex II activity in control and patient-derived ONS cells (D) Citrate synthase activity (E) Mitochondrial complex I activity after 50 nM rotenone treatment. Data are represented as Mean ± SD (n = 8 control vs. n = 8 patient-derived cells)

We next asked whether the rotenone sensitivity observed in patient-derived cells was due to pre-existing differences in mitochondrial complex I in ONS cells. We employed the standard mitochondrial isolation and the anti-TOM22 magnetic bead technique to isolate mitochondria and measure mitochondrial complex activities. Using mitochondrial preparations from these independent techniques, we observed that patient-derived cells had a significantly lower complex I activity compared to control-derived cells (Fig 5A and 5B) (-24.9% in isolated mitochondria and -23.8% in enriched mitochondrial preparations). Rotenone treatment (50nM) resulted in a 25.25% decrease in mitochondrial complex I activity in control-derived ONS cells and a 16.16% decrease in patient-derived cells. Therefore in the presence of rotenone mitochondrial complex I activity was 15.8% lower in patient-derived cells (Fig 5E). We measured complex II and citrate synthase activities as controls for differences in mitochondrial number. Both control-derived and patient-derived cells showed similar complex II and citrate synthase activity (Fig 5C and 5D) (0.70% and 0.04% change, respectively) indicating that there is a specific complex I deficit in patient-derived cells.

### Rotenone susceptibility phenotype is shared by genetic and idiopathic Parkinson’s disease patient-derived cells

**Fig 6:**
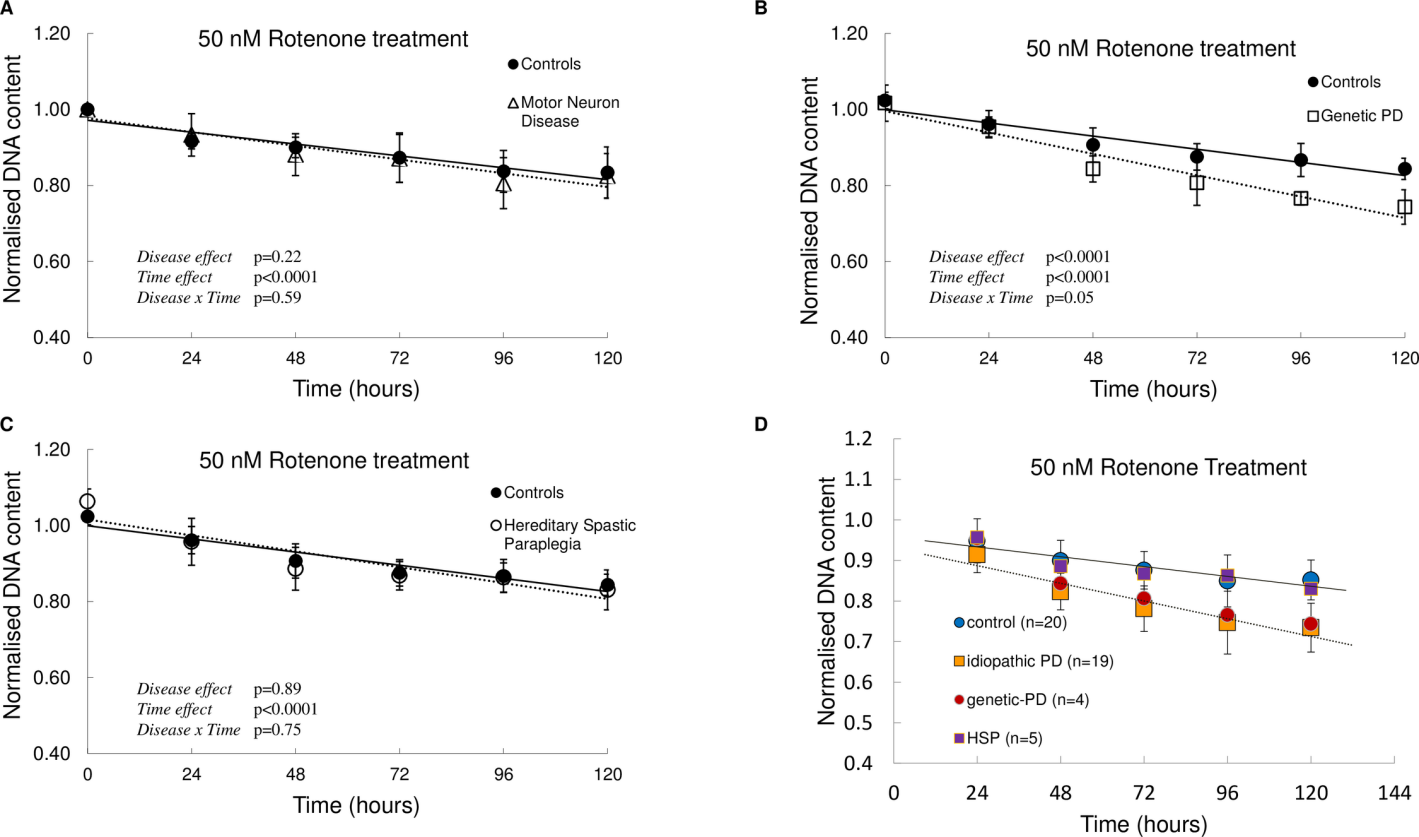
Effect of rotenone on ONS cells from patients with Motor Neuron Disease, Parkinson’s disease (idiopathic and monogenic), and Hereditary Spastic Paraplegia. (A, B, C) Cells were exposed to 50 nM Rotenone over 120 hours in culture and DNA content was determined every 24 hours by CyQUANT assay (n = 8 control, n = 5 MND, n = 4 genetic-PD and n = 5 HSP). (D) ONS cells from Parkinson’s disease patients (idiopathic and monogenic) are susceptible to Rotenone. Cells were exposed to 50 nM Rotenone over 120 hours in culture and DNA content was determined every 24hours by CyQUANT assay (n = 20 control, n = 19 idiopathic Parkinson’s disease, n = 4 genetic Parkinson’s disease (3 LRRK2 and 1 PINK1), n = 5 HSP). Data are represented as Mean ± SD. Solid line represents linear fit for controls and dotted line represents linear fit for patient cells. Graphs are annotated with p-values from a two-way ANOVA.

We next asked whether the rotenone susceptibility phenotype observed was relevant only to Parkinson’s disease ONS cells by assaying the response of ONS cells derived from patients with other neurodegenerative diseases. We exposed ONS cells derived from patients with other movement disorders including motor neuron disease (MND) and hereditary spastic paraplegia (HSP) to 50 nM rotenone. We observed that MND and HSP patient-derived cells and control-derived cells displayed similar sensitivity to rotenone (percentage change in MND patient-derived cells: 24 hours = -1.8%, 48 hours = -2.08%, 72 hours = -0.23%, 96 hours = -3.65%, 120 hours = -6.21%; in HSP patient-derived cells: 24 hours = -0.42%, 48 hours = -2.27%, 72 hours = -0.87%, 96 hours -0.46%, 120 hours = -1.6%). We also assayed the sensitivity of ONS cells from patients with monogenic forms of Parkinson’s disease, LRRK2 and PINK-1 mutations, in particular (percentage change at 24 hours = -0.71%, 48 hours = -6.94%, 72 hours = -7.84% 96 hours = -11.67% and 120 hours = -11.89%). Patient-derived cells from both idiopathic and monogenic forms of Parkinson’s disease showed increased susceptibility to rotenone (Fig 6C and 6D).

### Patient-derived cells have a diminished transcriptional response to rotenone

To further understand the molecular mechanisms underlying the rotenone susceptibility of Parkinson’s disease patient-derived ONS cells, we performed transcriptomic analysis on patient-derived and control-derived cells treated with rotenone or vehicle (DMSO) only. Principal component analysis revealed that rotenone treatment was the biggest driver of expression differences between the experimental groups (S5 Fig). In both control-derived and patient-derived cells, rotenone affected expression of genes encoding the enzyme complex of the electron transport chain of mitochondria (S6 Fig). However, no disease-specific differences were observed. Expression differences of cell death-associated genes were observed for proteins across multiple cellular compartments, from the extracellular space to the nucleus (S7 Fig).

**Fig 7:**
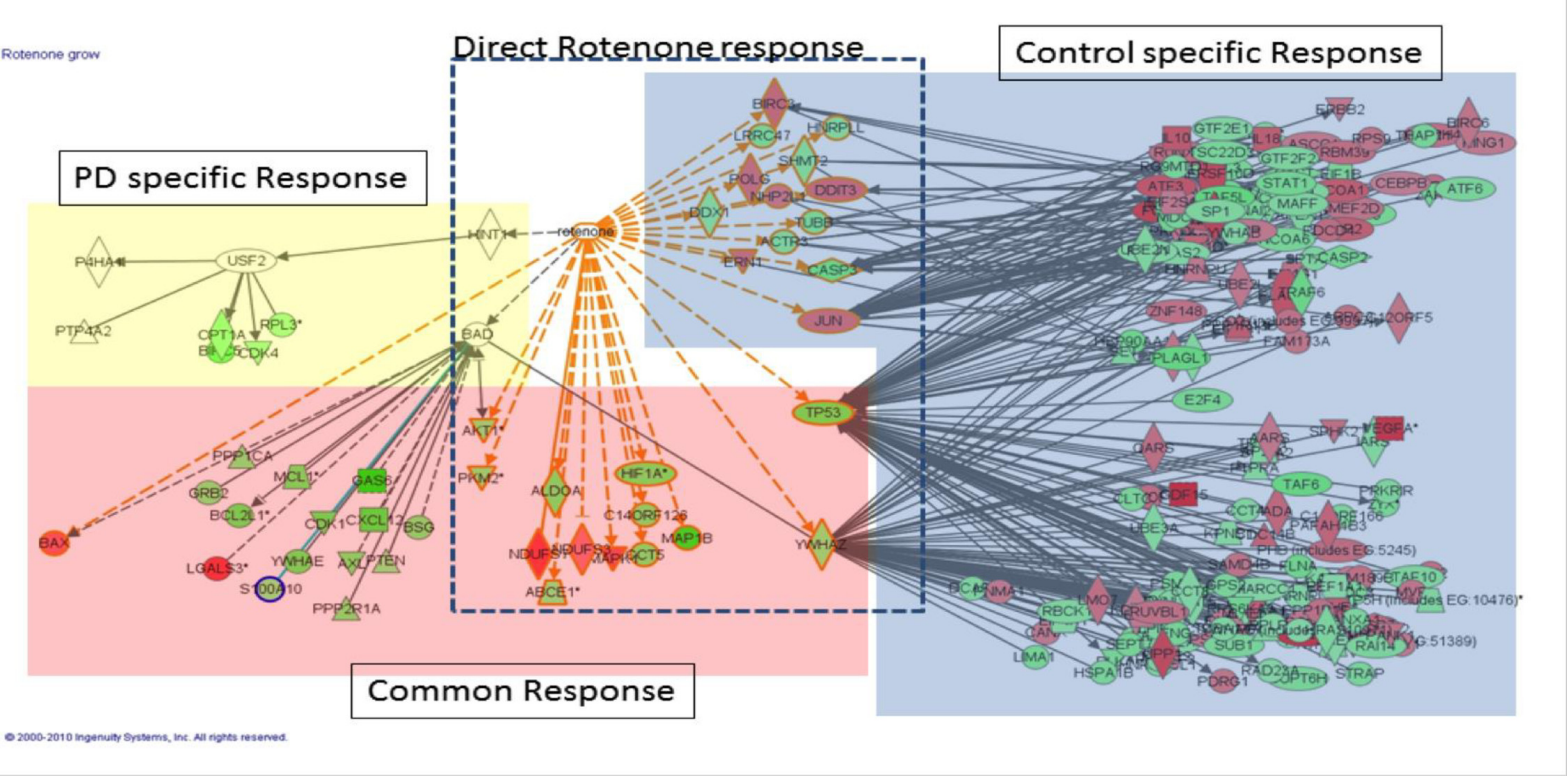
Patient-derived cells show a diminished transcriptional response after rotenone exposure. (A) Graphical representation of the Rotenone induced response in control and patient-derived cells. Ingenuity Pathway annotation mRNA set. Induced mRNAs in red and repressed in green.

The most striking observation was a drastically diminished transcriptional response in patient-derived cells compared to controls in response to rotenone treatment (Fig 7). In principle this could be attributable to two, not mutually exclusive, scenarios. Either exposure of patient-derived cells to rotenone (i) up-regulated the expression of factors, which actively induced apoptosis and/or (ii) failed to induce signals which protected against stress and apoptosis. Our data (Fig 7) supports the second explanation, as genes opposing apoptosis (BIRC3), responding to stress (DDIT3, ERN1) or promoting mitochondrial DNA synthesis (POLG) were only up-regulated in control cells. Concomitant suppression of genes regulating RNA metabolism (LRRC47, HNRPLL, DDX1), and execution of apoptosis (CASP3) was observed in control but not patient-derived cells. No “direct rotenone response” gene was differentially expressed in the patient-derived cells suggesting that it is the paucity of the response in patient-derived cells, which underlies their susceptibility.

## Discussion

In this study, we demonstrate that 1) Parkinson’s disease patient-derived cells are more sensitive to mitochondrial stressors (rotenone, piericidin A and oxidative stress) but not to other cell stressors; 2) the sensitivity to rotenone is observed in cells from patients with idiopathic Parkinson’s disease as well as some genetic forms Parkinson’s disease, but not in cells from patients with other neurodegenerative diseases; 3) patient-derived cells have lower mitochondrial complex I activity and, 4) patient-derived cells have a diminished transcriptional response to rotenone exposure.

Mitochondrial dysfunction has been implicated in the pathogenesis of both sporadic and genetic forms of Parkinsons’ disease [8,10,16,31]. The disruption of oxidative phosphorylation, particularly mitochondrial complex I disruption, has been linked to the loss of neurons in Parkinson’s disease [34,37,38]. A decrease in complex I activity has also been reported in peripheral tissues of Parkinson’s disease patients such as platelets, skeletal muscle and skin-derived fibroblasts [39–44]. Peripheral tissue derived models of Parkinson’s disease have been invaluable in understanding the molecular mechanisms underlying idiopathic Parkinson’s disease. Human skin fibroblasts have been used widely due to their ease of availability and their ability to mirror risk factors associated with specific individuals [45]. However, the results of measuring mitochondrial complexes from sporadic Parkinson’s disease patients are not consistent. For instance, one study [46] reported a reduction in mitochondrial complex I that could be rescued by coenzyme Q10 in a subtype of fibroblasts while another reported no difference in complex I [20]. Regardless of the conflicting reports, it is now accepted that in many cases of Parkinson’s disease there is a decrease in complex I activity.

We previously established ONS cells as a suitable system to model some aspects of idiopathic Parkinson’s disease. We reported metabolic and functional differences in idiopathic Parkinson’s disease patient-derived ONS cells and identified oxidative stress, mitochondrial dysfunction and xenobiotic metabolism as the main signalling pathways altered [29,30]. Here, we identified an endogenous mitochondrial complex I deficiency in ONS cells derived from idiopathic Parkinson’s disease patients. We were able to observe ~25% complex I inhibition comparable to what has been reported for the brain, muscle and platelets of Parkinson’s disease patients [39–44]. The observation that peripheral mitochondrial complex I deficiency can be associated with a disease primarily affecting dopaminergic neurons suggests that the effective regulation of mitochondrial bioenergetics is important for the prevention of neurodegeneration in idiopathic Parkinson’s disease. In fact, studies from the rotenone model suggest that a mild systemic impairment of mitochondrial complex I is sufficient to cause many of the pathological and behavioural hallmarks of Parkinson’s disease [47–49]. Rotenone, a potent mitochondrial complex I inhibitor, has been linked to mitochondrial dysfunction in animal models of Parkinson’s disease and also in humans [10,47,50]. Here, we show that Parkinson’s disease patient-derived ONS cells are very sensitive to rotenone exposure and a low dose of 50 nM is sufficient to induce cell death. The reported IC50 for rotenone varies between 20 nm to 200 μm depending on the model system used [47,51–55]. Recently, Ambrosi et al., [56] reported bioenergetics and proteolytic defects in sporadic Parkinson’s disease patient derived fibroblast cell lines. In contrast to our results, they did not observe any differences in apoptosis after rotenone administration even at much higher concentrations (500 μm) of rotenone. High concentration of rotenone completely inhibits complex I activity and may lead to non-specific effects [57]. We show that our patient-derived ONS cells display remarkable (10,000 times) sensitivity to rotenone exposure compared to patient-derived fibroblasts. Our findings are also strengthened by the use of cell lines from a large patient (n = 19) and control (n = 19) cohort. We also observed that the susceptibility to Rotenone is not reflected in ONS cells derived from patients with MND and HSP patients, suggesting that this may be a unique feature of Parkinson’s disease.

A cell responds to stress, either intrinsic or extrinsic, in different ways that range from activation of pro-survival pathways to initiating apoptotic events for elimination of damaged cells. Therefore, the key to a cell’s survival is its ability to mount an appropriate stress response. Heat shock proteins (HSPs) are molecular chaperones they promote cell survival in response to cellular stress and maintain homeostasis [58]. The expression of small heat shock protein, HSP27, has been reported to correlate with increased cell survival in response to stressful stimuli. HSP27 negatively regulates pro-apoptotic factors such as cytochrome C and interacts with key components of the apoptosome, thereby, regulating caspase-dependent cell death [59,60]. Consistent with previous studies, we observed that in Parkinson’s disease patient-derived ONS cells exposed to rotenone, HSP27 levels were reduced, and overexpression of HSP27 confers a protective effect against rotenone-induced stress.

Our transcriptomics analysis of control-derived and patient-derived cells treated with rotenone revealed an overall paucity of response in patient-derived cells. Interestingly, these results parallel the microarray results following exposure of Parkinson’s disease patient and control ONS cells to L-Sulforaphane treatment and Nrf2 induction [29]. Our microarray data raises three main possibilities, namely that the increased susceptibility of patient-derived cells to rotenone treatment was due to 1) the presence of a Parkinson’s disease specific response 2) the failure to initiate rotenone response pathways observed in control-derived cells 3) differences in the levels of induction in the common response genes. None of these possibilities are mutually exclusive. Although we identified an endogenous deficit in mitochondrial complex I in patient-derived cells and a susceptibility to rotenone treatment, we did not observe any differences in the mitochondrial complex I gene expression levels in control-derived and patient-derived cells treated with Rotenone. Perhaps, the baseline complex I deficiency in patient-derived cells is a reflection of broader deregulation of cellular bioenergetics.

Future directions include, but are not limited to, *in vitro* differentiation of ONS cells into dopaminergic neurons and investigation of rotenone-susceptibility in these cell types, investigation of the functions of HSP27 in mitochondrial stress and regulation of other pathways. The manipulation of HSP27 as a therapeutic strategy is unlikely because HSP27 is a pleiotropic inhibitor of apoptosis [59,61–63]. However, identification of small molecules that selectively activate one pathway in patient-derived cells or induce HSP27 may prove very valuable.

Overall, our data links the causes of idiopathic Parkinson’s disease with the pathways implicated in some genetic forms and emphasise the primacy of mitochondrial function in this disease. It also raises the question of whether the cause of “idiopathic” Parkinson’s disease is due to unknown genetics, rather than due to unknown environmental exposures. We propose that the rotenone-susceptibility phenotype observed in idiopathic Parkinson’s disease patient-derived cells may be useful for high throughput screening and identification of potential biomarkers for idiopathic Parkinson’s disease.

## Supporting Information

S1 Fig CyQUANT levels change linearly with cell number. ONS cells were counted using a Beckman Coulter and seeded in 384-well plates. CyQUANT assay was carried out according to the standard protocol. The fluorescence emission was plotted against the cell number. The standard curve was linear (R^2^ = 0.987) and was able to detect cells ranging from 100–700 and 2000–6000 cells per well.
(TIF)

S2 Fig Dose response curves of cell stressors on relative cell number. Control-derived cells were exposed to Rotenone, Hydrogen Peroxide, Chloroquine, Camptothecin, Tunicamycin and Piericidin A. Relative cell number was measured using CyQUANT assay. Dose-response curves were generated to determine the optimal stressor concentrations. Red arrows indicate the dose that leads to a 15–20% reduction in cell number after 48 hours in culture. Data represented as Mean ± SD, n = 3
(TIF)

S3 Fig Rate of H_2_O_2_ degradation in ONS cell culture medium. (TIF)

S4 Fig Apoptosis analysis of control and patient-derived ONS cells after Rotenone treatment. Cells were treated with Rotenone at 50 nM for 48 hours and CyQUANT, ViaLight, ToxiLight, Multi-Tox and Caspase 3/7 activities were measured. Data are represented as Mean ± SD, n = 8 Control vs. n = 8 Patient-derived
(TIF)

S5 Fig 3D Principal component analysis showing treatment with rotenone is the biggest driver of expression differences. (TIF)

S6 Fig Mitochondrial dysfunction pathway from Ingenuity Pathway analysis. Red = genes upregulated by rotenone and Green = genes downregulated by rotenone
(TIF)

S7 Fig Molecules in GO category ‘Cell death’ that are differentially expressed between patient-derived and control cells after rotenone treatment. Red = upregulated in patient-derived cells after rotenone treatment and Green = downregulated in patient-derived cells after rotenone treatment.
(TIF)

S8 Fig Apoptosis array for multiple apoptosis related proteins in control and patient-derived cells treated with 50 nM Rotenone for 48 hours (n = 8 control vs. n = 8 patient-derived) (TIF)

S1 Table Cell Line ID and data of subjects involved in the study. (DOCX)

S2 Table Percent change in patient-derived cells after exposure to stressors. (DOCX)
