# Supplementary figures and images for: Rotenone Susceptibility Phenotype in Olfactory Derived Patient Cells as a Model of Idiopathic Parkinson’s Disease
Source: PLoS One. 2016 Apr 28;11(4):e0154544. doi: 10.1371/journal.pone.0154544 (PMC4849794; doi:10.1371/journal.pone.0154544)

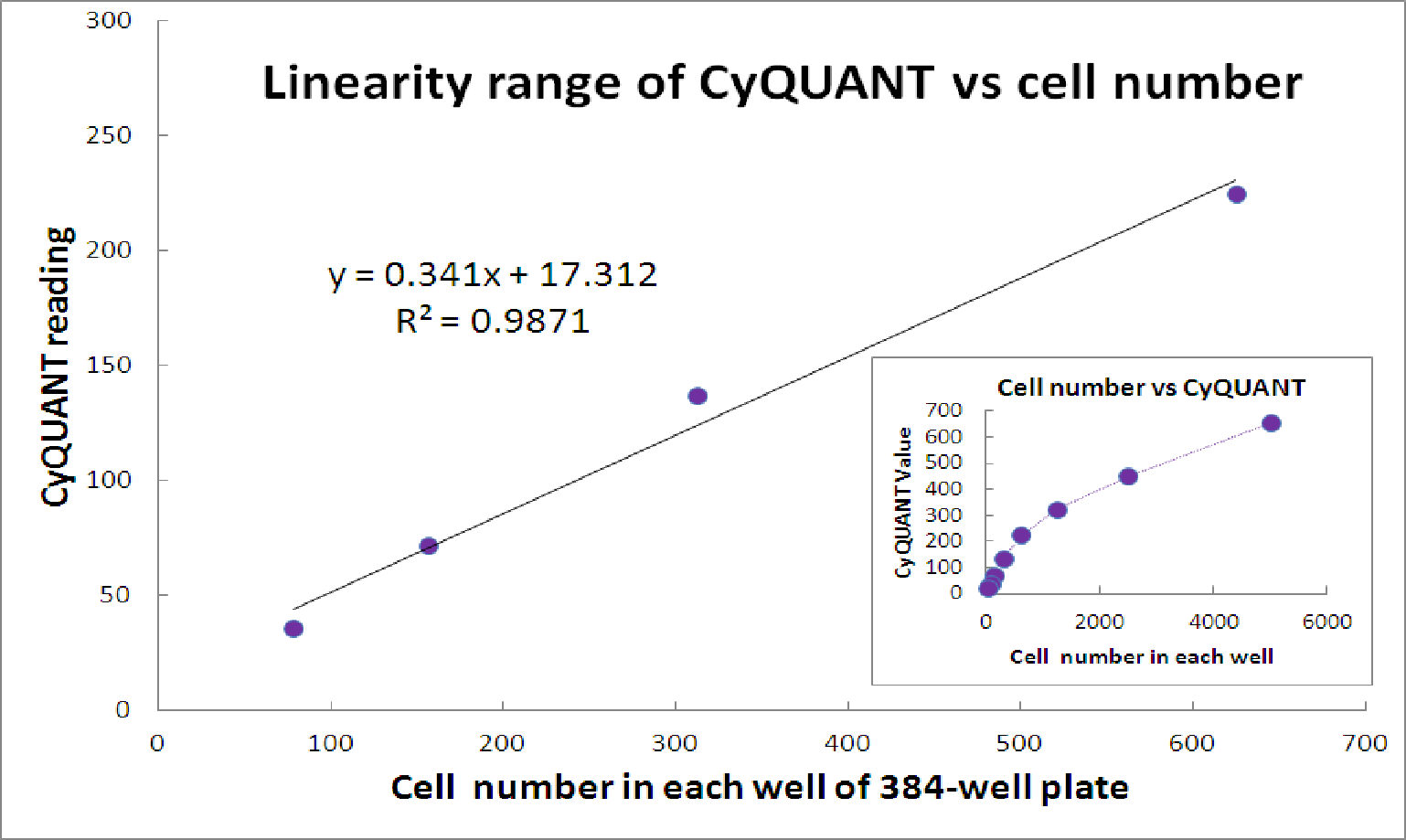

Supplement: S1 Fig — ONS cells were counted using a Beckman Coulter and seeded in 384-well plates. CyQUANT assay was carried out according to the standard protocol. The fluorescence emission was plotted against the cell number. The standard curve was linear (R2 = 0.987) and was able to detect cells ranging from 100–700 and 2000–6000 cells per well. (TIF) [file pone.0154544.s001.tif]

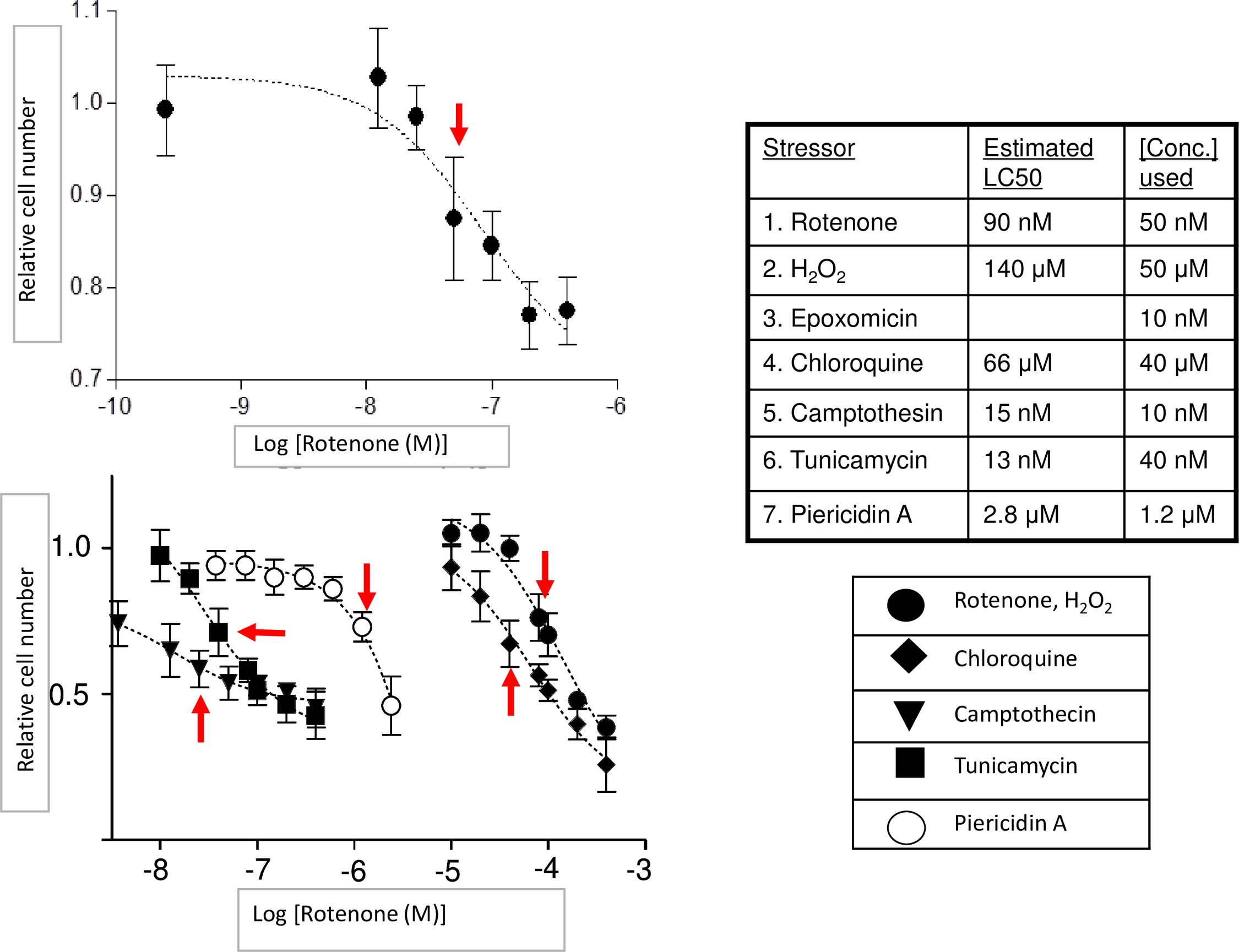

Supplement: S2 Fig — Control-derived cells were exposed to Rotenone, Hydrogen Peroxide, Chloroquine, Camptothecin, Tunicamycin and Piericidin A. Relative cell number was measured using CyQUANT assay. Dose-response curves were generated to determine the optimal stressor concentrations. Red arrows indicate the dose that leads to a 15–20% reduction in cell number after 48 hours in culture. Data represented as Mean ± SD, n = 3 (TIF) [file pone.0154544.s002.tif]

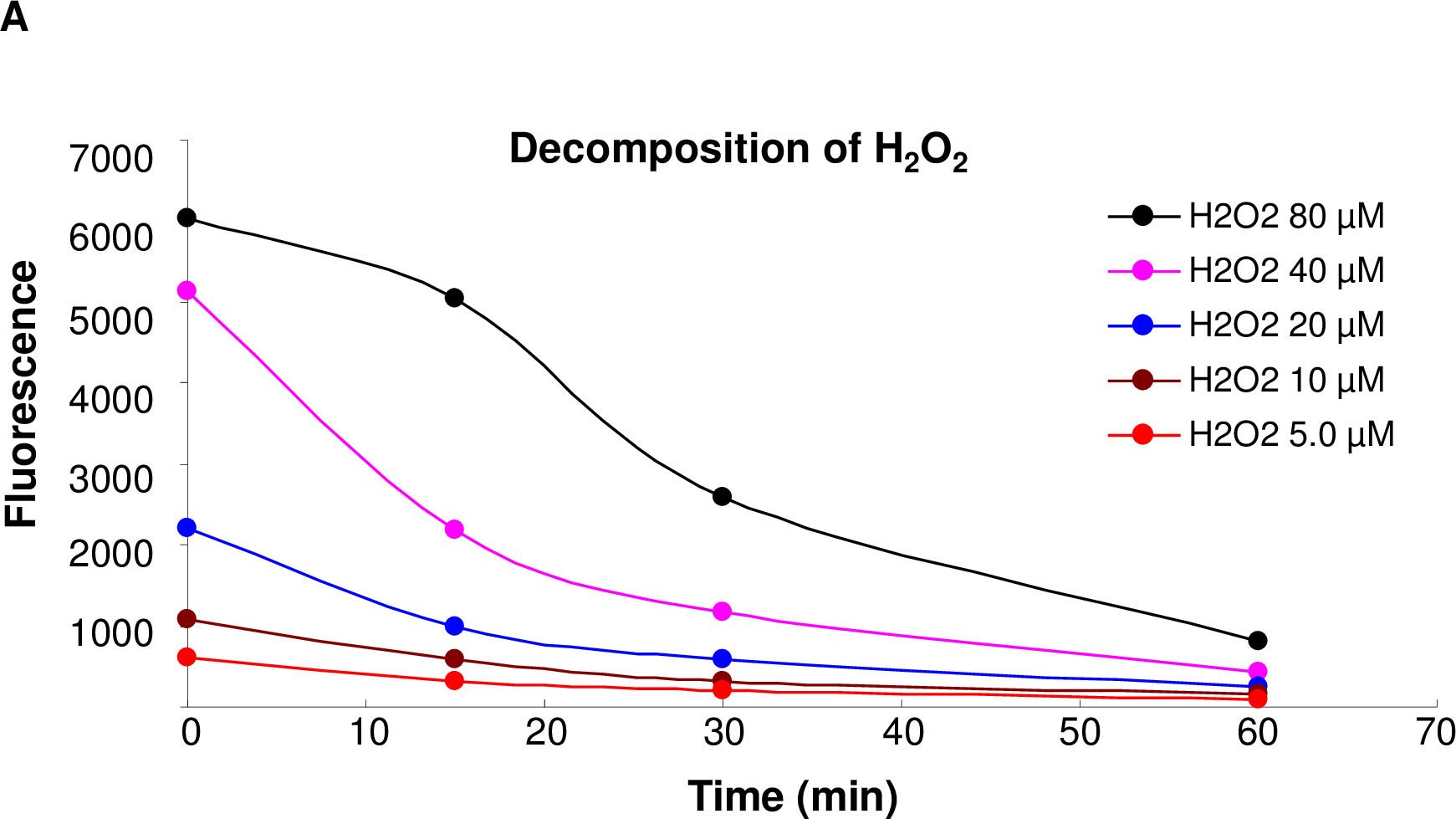

Supplement: S3 Fig — (TIF) [file pone.0154544.s003.tif]

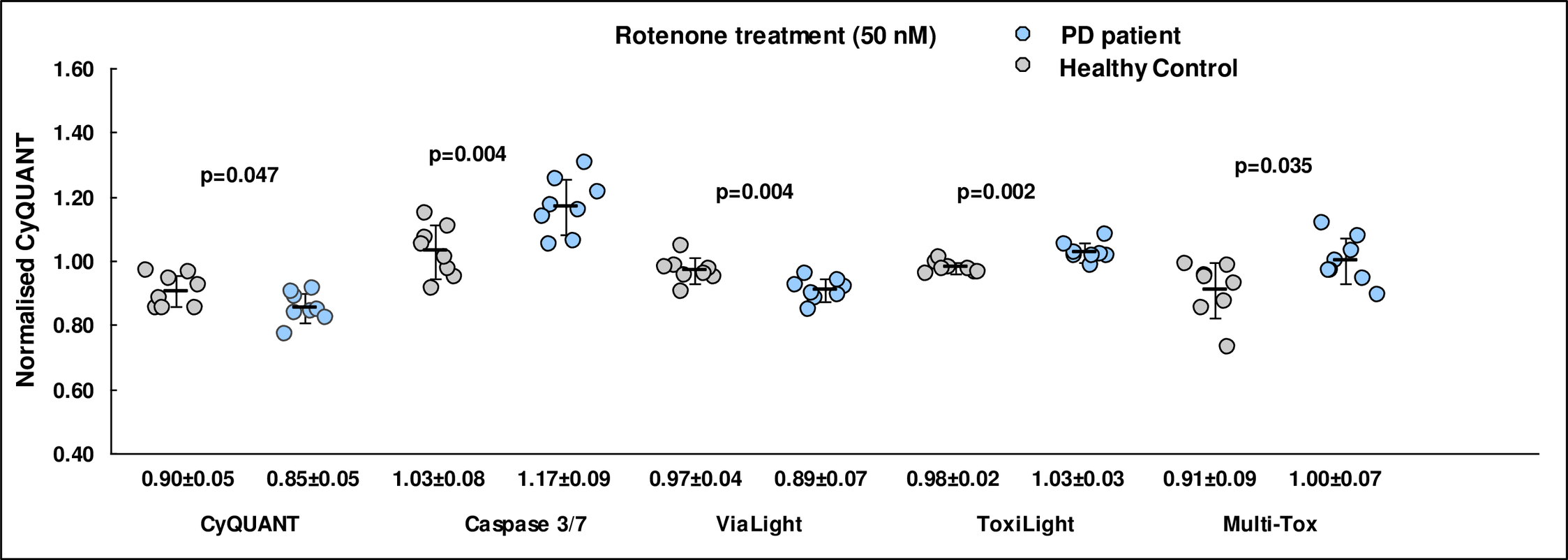

Supplement: S4 Fig — Cells were treated with Rotenone at 50 nM for 48 hours and CyQUANT, ViaLight, ToxiLight, Multi-Tox and Caspase 3/7 activities were measured. Data are represented as Mean ± SD, n = 8 Control vs. n = 8 Patient-derived (TIF) [file pone.0154544.s004.tif]

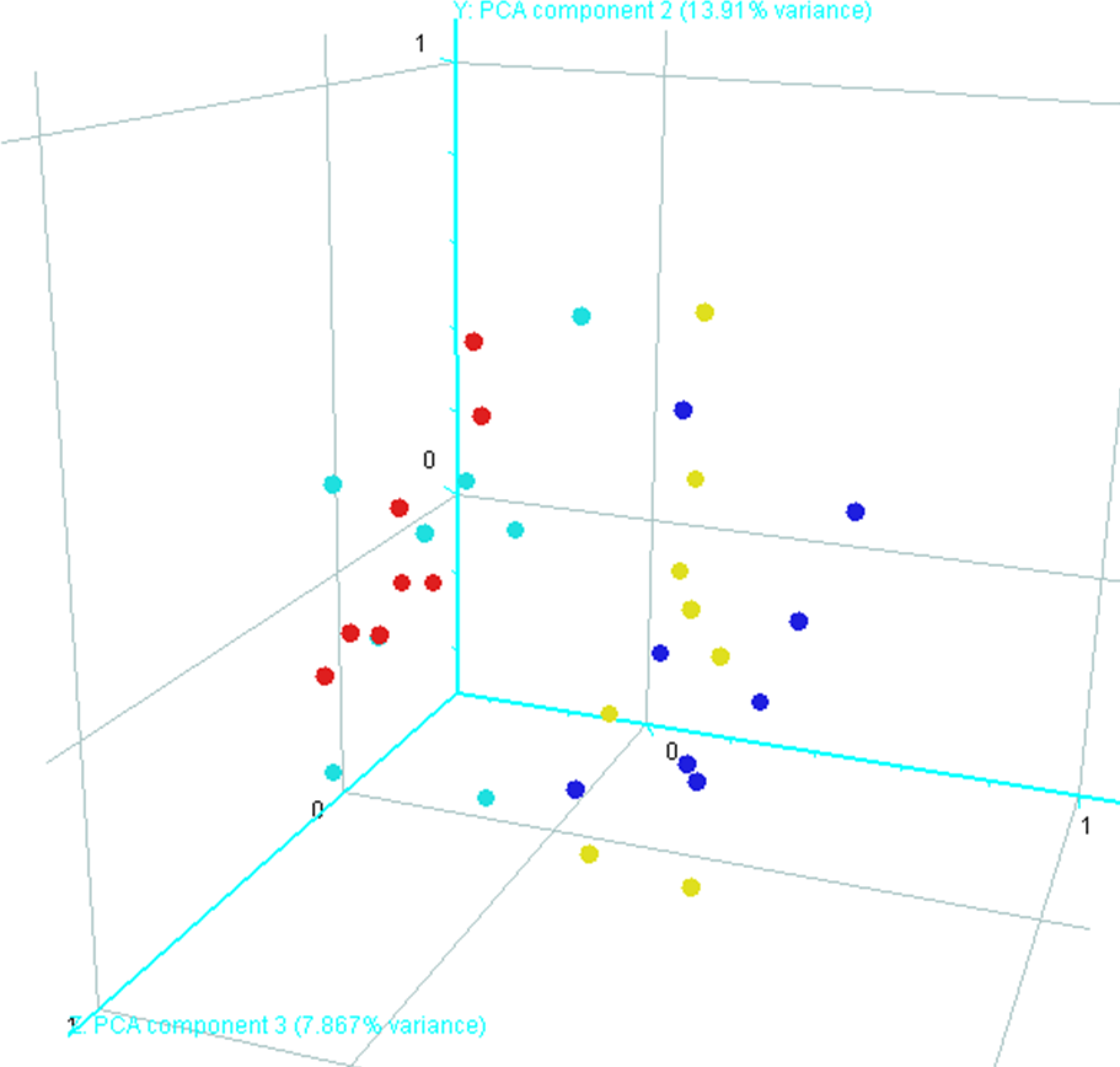

Supplement: S5 Fig — (TIF) [file pone.0154544.s005.tif]

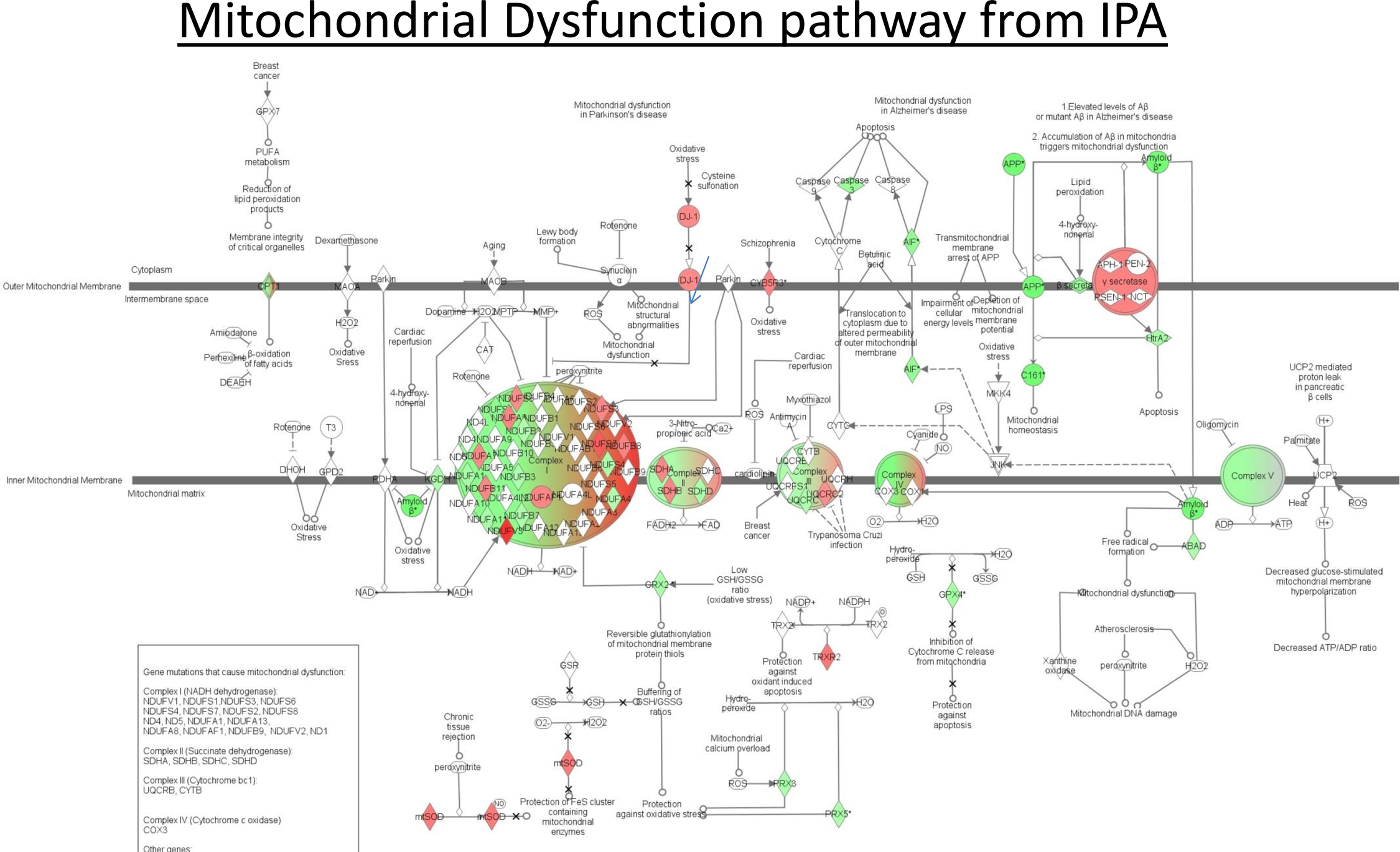

Supplement: S6 Fig — Red = genes upregulated by rotenone and Green = genes downregulated by rotenone (TIF) [file pone.0154544.s006.tif]

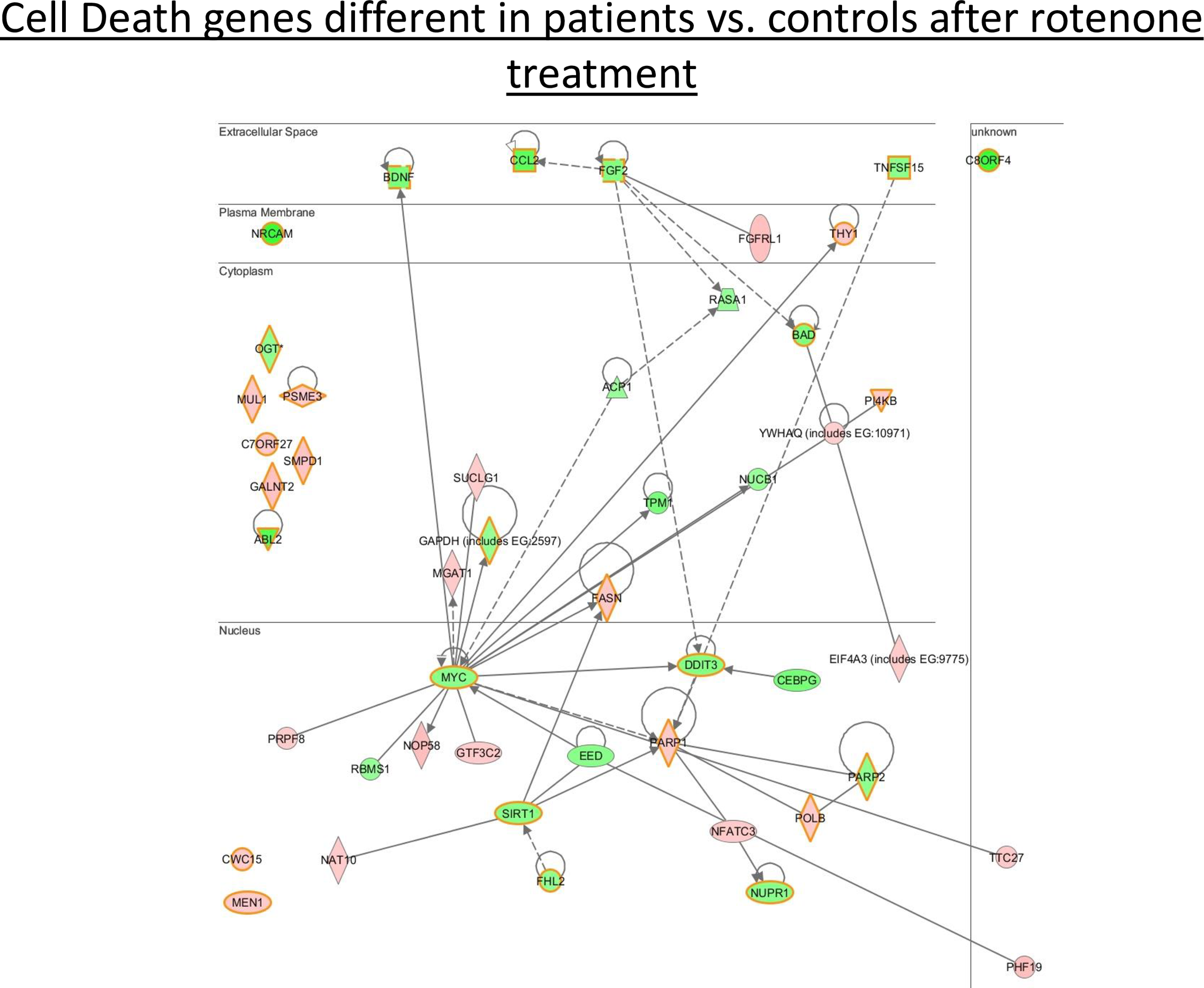

Supplement: S7 Fig — Red = upregulated in patient-derived cells after rotenone treatment and Green = downregulated in patient-derived cells after rotenone treatment. (TIF) [file pone.0154544.s007.tif]

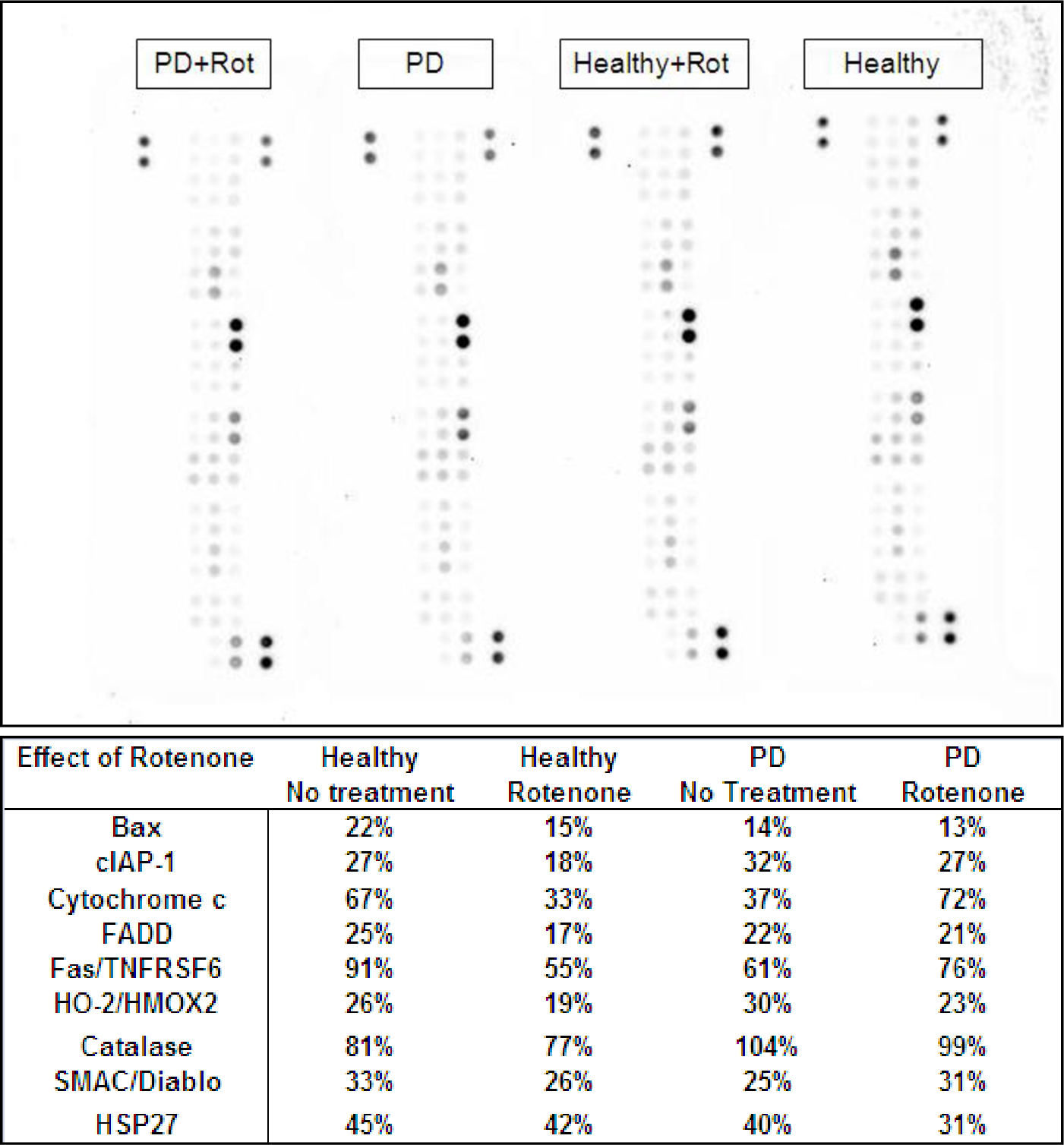

Supplement: S8 Fig — (TIF) [file pone.0154544.s008.tif]
