# Supplementary material for: Rotenone Susceptibility Phenotype in Olfactory Derived Patient Cells as a Model of Idiopathic Parkinson’s Disease
Source: PLoS One. 2016 Apr 28;11(4):e0154544. doi: 10.1371/journal.pone.0154544 (PMC4849794; doi:10.1371/journal.pone.0154544)
Supplement: S1 Table — (DOCX) [file pone.0154544.s009.docx]

S1 Table: Cell Line ID and data of subjects involved in the study

| **Cell Line ID** | **Age** | **Sex** |
| --- | --- | --- |
| Control | | |
| 100080017 | 49 | Male |
| 100080009 | 61 | Male |
| 100090001 | 70 | Female |
| 100080015 | 64 | Female |
| 100080016 | 60 | Male |
| 100080004 | 59 | Female |
| 100080003 | 66 | Female |
| 100080002 | 59 | Male |
| 100030011 | 31 | Female |
| 100030012 | 40 | Male |
| 100030013 | 72 | Female |
| 100030014 | 47 | Male |
| 100040003 | 29 | Male |
| 100040005 | 25 | Female |
| 100080012 | 64 | Male |
| 100080014 | 65 | Female |
| 100080013 | 64 | Male |
| 100090004 | 37 | Female |
| 100090001 | 70 | Female |
| 100090003 | 27 | Female |
| Sporadic Parkinson’s disease | | |
| 200080001 | 57 | Female |
| 200080007 | 57 | Male |
| 200080013 | 58 | Female |
| 200080015 | 54 | Male |
| 200080017 | 61 | Male |
| 200080020 | 63 | Female |
| 200080021 | 61 | Male |
| 200080022 | 54 | Male |
| 200060004 | 67 | Female |
| 200070001 | 47 | Female |
| 200070002 | 80 | Male |
| 200070003 | 52 | Female |
| 200070005 | 26 | Male |
| 200090004 | 61 | Male |
| 200090001 | 56 | Male |
| 200090002 | 31 | Female |
| 200090010 | 30 | Female |
| 200080023 | 79 | Male |
| 200050008 | 70 | Female |
| 200050009 |  | Male |
| Genetic forms of Parkinson’s disease | | |
| 203060001 (PINK) | 65 | Female |
| 201550001 (LRRK2,1441) | 66 | Male |
| 202550001 (LRRK2, 2019) | 78 | Female |
| 202060001 (LRRK2, 2019) | 69 | Male |
| Hereditary Spastic Paraplegia | | |
| 610080001 | 46 | Female |
| 610080002 | 50 | Female |
| 610080005 | 32 | Female |
| 610080007 | 51 | Female |
| 610080008 | 57 | Male |
| Motor Neuron Disease | | |
| 500070001 | 40 | Male |
| 500070002 | 37 | Male |
| 500080001 | 45 | Male |
| 500080002 | 45 | Male |
| 500080003 | 46 | Male |
