# Supplementary material for: Rotenone Susceptibility Phenotype in Olfactory Derived Patient Cells as a Model of Idiopathic Parkinson’s Disease
Source: PLoS One. 2016 Apr 28;11(4):e0154544. doi: 10.1371/journal.pone.0154544 (PMC4849794; doi:10.1371/journal.pone.0154544)
Supplement: S2 Table — (DOCX) [file pone.0154544.s010.docx]

S2 Table: Percent change in patient-derived cells after exposure to stressors

| Stressor | Time in hours | | | | |
| --- | --- | --- | --- | --- | --- |
|  | 24 | 48 | 72 | 96 | 120 |
| Epoxomicin | 1.257862 | 2.439024 | -3.80952 | -1.79211 | 3.781513 |
| Chloroquine | -0.10965 | 3.469641 | -2.46753 | -0.68027 | -2.23152 |
| Camptothecin | -7.08955 | -4.56942 | 3.030303 | 0.378072 | 0 |
| Tunicamycin | 1.837482 | 4.367313 | 2.098565 | 5.436958 | 3.820461 |
